# Type II A aortic dissection and ascending aort aneurysm seeing after mechanic aortic valve replacement

**DOI:** 10.1186/1749-8090-10-S1-A111

**Published:** 2015-12-16

**Authors:** Kazim Ergunes, Levent Yilik, Ihsan Peker, Koksal Donmez, Ali Gurbuz

**Affiliations:** 1 Katip Celebi University Ataturk Training and Research Hospital, Cardiac and Vascular Surgery Clinic, Izmir, Turkey

## Background/Introduction

Type II A aortic dissection after mechanic aortic valve replacement is a major complication and may be life threatening.

## Aims/Objectives

We present a case of a 51 years-old male patient having an acute Type II A aortic dissection after mechanic aortic valve replacement.

## Method

A 51-years-old male was hospitalized in our clinic on Mach 26, 2015. He had mild aortic insufficiency and mild mitral insufficiency. Ejection fraction was 60%. CT-Scan revealed type II A aortic dissection.

## Results

He had Type II A aortic dissection with intimal tear at proximal region of the right coronary ostium. Mechanic aortic valve was normal function. Ascending aorta was replaced by using 26 mm Dacron tube graft between annulus of previous mechanic aortic valve and distal ascending aort by using hypothermic circulatory arrest and antegrade selective cerebral perfusion. An 8 mm dacron tube graft was anastomosed between right and left coronary annulus and this tube graft was anastomosed to neo-aorta (Cabrol technique). Postoperative period was event-free. The patient was discharged on 14 postoperative day with warfaine and methoprolol treatment.

## Discussion/Conclusion

Although acute aortic dissection after mechanic aortic valve replacement is a rare complication, physicians should keep this potential life-threatening complication in mind.

## Consent

Written informed consent was obtained from the patient for publication of this abstract and any accompanying images. A copy of the written consent is available for review by the Editor of this journal.

